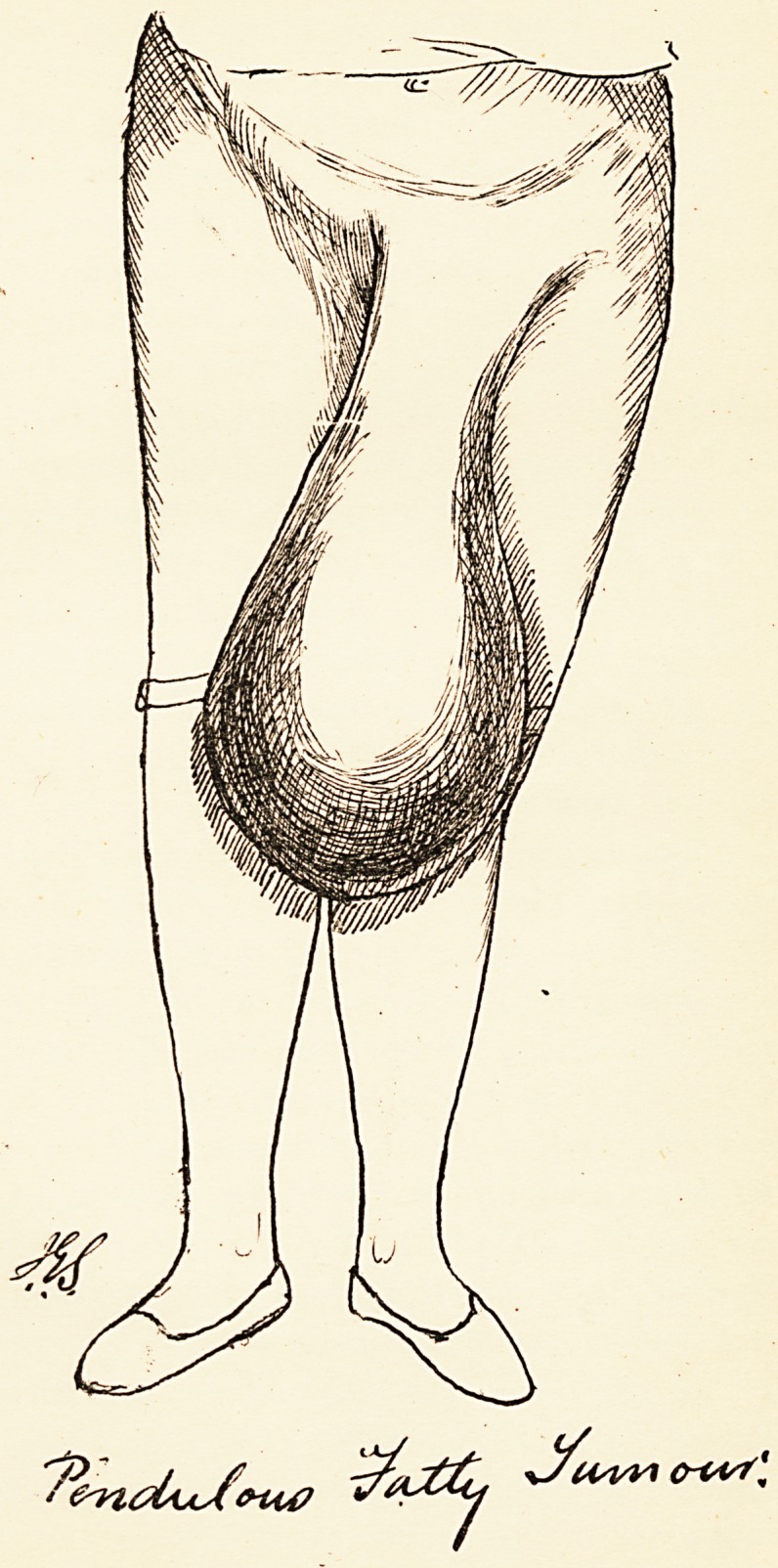# Recurrent Pendulous Fatty Tumour of Thigh

**Published:** 1883-07

**Authors:** 


### RECURRENT PENDULOUS FATTY TUMOUR OF THIGH. By the EDITOR.

J. I., æt. 66, a shoemaker in Cardiff, was admitted to the Bristol Royal Infirmary on April 21st, 1883, with a pendulous fatty tumour springing from the upper and inner aspect of the left thigh. Ten years previously a tumour of the same nature had been removed from the

Plate VII.

Recurrent Pendulous Fatty Tumour

Plate VIII.

*Pendulous Fatty Tumour.*

same situation in the Infirmary. Two years after this operation the tumour began to grow again under the scar, and had gradually increased up to date, becoming in the last eighteen months pendulous. The surface on the under and posterior aspect had begun to ulcerate two months prior to admission. The situation and size are shown in the accompanying plate, traced from a photograph. (Pl. VII.) It was removed, and the wound healed by first intention.

*Remarks.*—It seems extraordinary that the only other case of recurrent pendulous fatty tumour which I can find recorded should have been of nearly the same size and *in almost exactly the same situation.* The case is described by Mr. Curling in *Path. Trans.*, vol. xviii., p. 86.

"In May, 1863, I exhibited at a meeting of the society a large lobulated fatty tumour weighing upwards of a pound, which I had removed in that month from the left side of the scrotum, lower part of the abdomen and inner part of the left thigh of a gentleman who had undergone three previous operations; the first by Mr. Lawrence, in 1845; the others by myself, for fatty tumours in the same part of the body. About two years after this last operation the patient called and showed me a pendulous tumour, about the size of a hen's egg, growing from the inner part of the thigh near the cruro-scrotal fold, and another swelling beneath the old cicatrix near the pubes. A fifth operation was performed on May 29th, 1866. After removing the pendulous growth I found and excised a well-defined oval fatty tumour, the size of a hen's egg, embedded in the muscles of the thigh beneath the pectineus. . . . . The chief point of pathological interest is the persistent tendency to the formation of adipose growth on the site of the original tumour and in the adjacent parts of the thigh. I know of no similar case of recurrent fatty tumour."

Report by Mr. De Morgan and Mr. Hulke on Mr. Curling's tumours:—"The specimens present for the most part the appearances met with in ordinary fatty tumours. There are, however, certain peculiarities in their mode of growth and in their structure. In place of being separated from the structure in which they are embedded by a sheath of connective tissue, so as to permit of their complete enucleation, or attached to them by a neck consisting of the same elements as the tumours themselves, they originate in a pedicle which has in all instances been cut through. This pedicle contains a large amount of delicate fibroid tissue, which towards its root is arranged in the form of bundles; these spread out and form at first small and then larger areolae in which the fat vesicles are deposited. . . . The constant occurrence of a peduncle, and the evidently active growth of connective tissue in this case, are in harmony with the recurrence of the tumours *in loco*."

In this connexion it will be interesting to note a case of pendulous fatty tumour which was operated upon in the Bristol Infirmary by Mr. Richard Smith in 1834. The patient was a woman aged 55, and *the tumour grew from the same situation.* It had been growing fourteen years and weighed six pounds. (Plate VIII.)

## Figures and Tables

**Figure f1:**
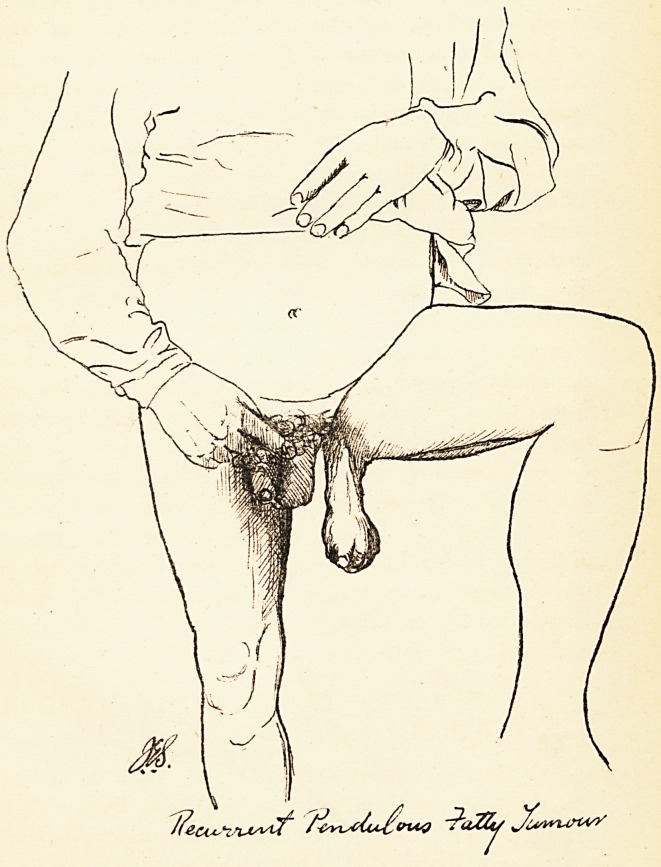


**Figure f2:**